# Appraisal of systemic inflammation and diagnostic markers in a porcine model of VAP: secondary analysis from a study on novel preventive strategies

**DOI:** 10.1186/s40635-018-0206-1

**Published:** 2018-10-20

**Authors:** Gianluigi Li Bassi, Raquel Guillamat Prats, Antonio Artigas, Eli Aguilera Xiol, Joan-Daniel Marti, Otavio T. Ranzani, Montserrat Rigol, Laia Fernandez, Andrea Meli, Denise Battaglini, Nestor Luque, Miguel Ferrer, Ignacio Martin-Loeches, Pedro Póvoa, Davide Chiumello, Paolo Pelosi, Antoni Torres

**Affiliations:** 1 Division of Animal Experimentation, Department of Pulmonary and Critical Care Medicine, Thorax Institute, Hospital Clinic, Calle Villarroel 170, Esc 6/8 Pl 2, Barcelona, Spain; 2 Institut d’Investigacions Biomèdiques August Pi i Sunyer (IDIBAPS), Barcelona, Spain; 3 Centro de Investigación Biomedica En Red- Enfermedades Respiratorias (CIBERES), Barcelona, Spain; 4 University of Barcelona, Barcelona, Spain; 5 Pathophysiological Laboratory, Institut de Investigacion Parc Tauli, Corporació Sanitaria Universitaria Parc Tauli, Autonomous University of Barcelona, Sabadell, Barcelona, Spain; 6 Cardiology Department, Hospital Clinic, Barcelona, Spain; 7 Dipartimento di Anestesia e Rianimazione, ASST Santi Paolo e Carlo, Dipartimento di Scienza e Salute, Universita degli Studi di Milano, Milan, Italy; 8 Dipartimento Scienze Chirurgiche e Diagnostiche Integrate (DISC), Università degli Studi di Genova, Genova, Italy; 9 Multidisciplinary Intensive Care Research Organization (MICRO), Department of Clinical Medicine, Trinity Centre for Health Sciences, St James’s University Hospital, Dublin, Ireland; 10 Polyvalent Intensive Care Unit, São Francisco Xavier Hospital, Centro Hospitalar de Lisboa Ocidental, Lisbon, Portugal; 11 NOVA Medical School, CEDOC, New University of Lisbon, Lisbon, Portugal

**Keywords:** Trendelenburg, Semirecumbent, Inflammation, Interleukin, Mechanical ventilation, Ventilator-associated pneumonia

## Abstract

**Background:**

We previously evaluated the efficacy of a ventilatory strategy to achieve expiratory flow bias and positive end-expiratory pressure (EFB + PEEP) or the Trendelenburg position (TP) for the prevention of ventilator-associated pneumonia (VAP). These preventive measures were aimed at improving mucus clearance and reducing pulmonary aspiration of bacteria-laden oropharyngeal secretions. This secondary analysis is aimed at evaluating the effects of aforementioned interventions on systemic inflammation and to substantiate the value of clinical parameters and cytokines in the diagnosis of VAP.

**Methods:**

Twenty female pigs were randomized to be positioned in the semirecumbent/prone position, and ventilated with duty cycle 0.33 and without PEEP (control); positioned as in the control group, PEEP 5 cmH_2_O, and duty cycle to achieve expiratory flow bias (EFB+PEEP); ventilated as in the control group, but in the Trendelenburg position (Trendelenburg). Following randomization, *P. aeruginosa* was instilled into the oropharynx. Systemic cytokines and tracheal secretions *P. aeruginosa* concentration were quantified every 24h. Lung biopsies were collected for microbiological confirmation of VAP.

**Results:**

In the control, EFB + PEEP, and Trendelenburg groups, lung tissue *Pseudomonas aeruginosa* concentration was 2.4 ± 1.5, 1.9 ± 2.1, and 0.3 ± 0.6 log cfu/mL, respectively (*p* = 0.020). Whereas, it was 2.4 ± 1.9 and 0.6 ± 0.9 log cfu/mL in animals with or without VAP (*p* < 0.001). Lower levels of interleukin (IL)-1β (*p* = 0.021), IL-1RA (*p* < 0.001), IL-4 (*p* = 0.005), IL-8 (*p* = 0.008), and IL-18 (*p* = 0.050) were found in Trendelenburg animals. VAP increased IL-10 (*p* = 0.035), tumor necrosis factor-α (*p* = 0.041), and endotracheal aspirate (ETA) *P*. *aeruginosa* concentration (*p* = 0.024). A model comprising ETA bacterial burden, IL-10, and TNF-α yielded moderate discrimination for the diagnosis of VAP (area of the receiver operating curve 0.82, 95% CI 0.61–1.00).

**Conclusions:**

Our findings demonstrate anti-inflammatory effects associated with the Trendelenburg position. In this reliable model of VAP, ETA culture showed good diagnostic accuracy, whereas systemic IL-10 and TNF-α marginally improved accuracy. Further clinical studies will be necessary to confirm clinical value of the Trendelenburg position as a measure to hinder inflammation during mechanical ventilation and significance of systemic IL-10 and TNF-α in the diagnosis of VAP.

## Background

Ventilator-associated pneumonia (VAP) is a common iatrogenic pulmonary complication in critically ill patients on mechanical ventilation (MV) [1–3]. Clinical presentation of VAP is highly heterogenous ranging from mild to highly severe [4], potentially causing a systemic cytokine storm and septic shock [5]. Many efforts have been made to fully characterize pathophysiology of the disease and the host inflammatory response, improve diagnostic accuracy, and develop efficacious preventive strategies [2, 3].

Among the available preventive interventions [6], body position plays a critical role. Currently, intensive care unit (ICU) patients are kept with the head of the bed oriented above 30° to avoid gastro-pulmonary aspiration, namely the semirecumbent position [7]. A recent clinical trial has also tested preventive efficacy of the Trendelenburg position, which limits gravity-driven aspiration of oropharyngeal secretions [8]. Nevertheless, to date, the effects of body position on the host inflammatory response and potential association with the development of VAP are still unknown.

Inflammatory biomarkers in blood or bronchoalveolar lavage fluids of patients with VAP [9–11] not only have been tested to characterize inflammation but also to accurately and promptly diagnose VAP. Indeed, VAP is currently diagnosed using clinical criteria and microbiology cultures, which yield low specificity/sensitivity and are often too slow for clinical needs [12]. Unfortunately, aforementioned clinical studies were biased by the well-recognized challenges in VAP diagnosis, the extreme heterogeneity among ICU populations and degrees of severity. As a result, VAP still lacks a clinical diagnostic gold-standard.

We previously developed a reliable animal model of VAP [13] to circumvent some of aforementioned limitations encountered in clinical settings and to specifically evaluate novel diagnostic and preventive strategies. This model was recently used to study efficacy of (1) inverse-ratio ventilation with positive end-expiratory pressure (PEEP) or (2) the Trendelenburg position in the prevention of VAP. These ventilatory settings were applied because mucus clearance is enhanced through inverse-ratio ventilation [14, 15] and the Trendelenburg position [16], while gravity-driven pulmonary aspiration is reduced through PEEP [17].

## Methods

### Aim, design, and settings

We performed a secondary analysis of a previous study [18], conducted at the Division of Animal Experimentation, University of Barcelona, Barcelona, Spain. The primary goals of this secondary analysis were to evaluate dynamics of inflammatory biomarkers during application of novel VAP preventive strategies and to ascertain significance of various clinical parameters and cytokines in the diagnosis of VAP. Animals were managed according to the local guidelines and regulations for the use and care of animals. The animal experimentation ethical committee reviewed and approved the study protocol. Additional details on animal handling and methods are reported in previous publications [13, 18].

### Animal preparation and handling

We studied 21 Large White-Landrace female pigs, orotracheally intubated and mechanically ventilated. Animals were anesthetized and paralyzed. Endogenous pneumonia was prevented with ceftriaxone. The femoral artery and jugular vein were cannulated for hemodynamic monitoring and blood sampling.

### Clinical parameters

Body temperature, white blood cell count, and arterial partial pressure of oxygen were assessed before bacterial challenge and at 24, 48, and 72 h thereafter. The arterial partial pressure of oxygen per inspiratory fraction of oxygen ratio (PaO_2_/F_I_O_2_) was computed. At the same time points, serum creatinine and alanine transaminase were measured. Of note, reference values of aforementioned parameters in pigs are similar to those in humans. Finally, at 72 h, we collected tracheal secretions for quantitative microbiology culture, we qualitatively evaluated purulence, and we computed the clinical pulmonary infection score (CPIS), as described in Table 1.

**Table 1:** Clinical pulmonary infection score

CPIS points	0	1	2
Tracheal secretions	Rare	Abundant	Abundant and purulent
Chest radiograph infiltrates	No infiltrate	Disseminated	Localized
Temperature (°C)	≥ 36.5 and ≤ 38.4	≥ 38.5 and ≤ 38.9	≥ 39 and ≤ 36
Leukocytes count (10^3^/μl)	≥ 4 and ≤ 11	< 4 or > 11	
Pa_O2_/F_IO2_ (mmHg)	≥ 240		≤ 240
Microbiology	Negative		Positive

*CPIS* clinical pulmonary infection score. A CPIS score value ≥ 6 was considered suggestive of pneumonia. Chest radiographs were not collected. Nevertheless, given the initial healthy status of the animal and the macroscopic lung examination upon autopsy, we assumed in all pigs localized chest radiograph infiltrates in case of confirmed pulmonary infiltrates upon autopsy

### Randomization

Following surgical preparation, pigs were randomized as follows:

1. Control: Pigs were placed in prone position and ventilated as reported above, but without PEEP. As previously reported [17, 19], the surgical bed was oriented approximately 30° in the anti-Trendelenburg position to achieve an orientation of the respiratory system as in the semirecumbent position in humans.
2. Expiratory flow bias and PEEP (EFB + PEEP): Pigs were positioned as in the control group. The duty cycle (T_I_/T_TOT_) was adjusted daily to achieve a mean expiratory-inspiratory flow bias of 10 L/min and PEEP was set at 5 cm H_2_O. As previously described [17], this ventilatory strategy was aimed at improving mucus clearance through the resulting expiratory flow bias [14], and hindering pulmonary aspiration of colonized subglottic secretions through PEEP.
3. Trendelenburg: Pigs were in prone position and ventilated as in the control group. The surgical bed was oriented 5° below horizontal

To achieve aforementioned ventilatory endpoints, airway pressure was measured proximally to the endotracheal tube with a pressure transducer (MPX 2010 DP; Motorola, Phoenix, AZ, USA). Respiratory flow rates were measured with a heated pneumotachograph (Fleisch no. 2; Fleisch, Lausanne, Switzerland). Flow and pressure signals were recorded on a personal computer and assessed with dedicated software (Colligo; Elekton, Milan, Italy).

### Bacterial challenge

Shortly after randomization, 5 mL of 10^7^–10^8^ ceftriaxone-resistant *Pseudomonas aeruginosa* suspension was instilled into the oropharynx to colonize the oropharynx and promote aspiration of *P*. *aeruginosa*-laden oropharyngeal secretions and VAP [13].

### Systemic biomarkers

Prior to bacterial challenge, and at 24, 48, and 72 h thereafter, blood was drawn for measurement of serum inflammatory markers. Blood was centrifuged at 3000 rpm at 4 °C for 15 min, and serum aliquots were stored at − 80 °C. Serum interferon (INF)-γ; interleukin (IL)-1α; IL-1β; IL-1 receptor antagonist (RA); IL-2; IL-4; IL-6; IL-8; IL-10; IL-12; IL-18; and tumor necrosis factor (TNF)-α were quantified by bead-based multiplex assays with Luminex xMAP® technology (Millipore Iberica, S.A., Madrid, Spain). Whereas tissue factor, angiotensin-2, adrenomedullin, and protein C-reactive protein were quantified through enzyme-linked immunosorbent assay (ELISA) (Bionova cientifica S.L., Madrid, Spain). Accuracy in cytokine quantification by Luminex xMAP® technology is comparable to the ELISA assay [20–22]. Nevertheless, Luminex xMAP® assay allows measurement of multiple cytokines simultaneously providing additional benefits. All inflammatory markers data are reported as log pg/L. Aforementioned biomarkers were chosen based on previous clinical studies assessing systemic and pulmonary inflammation during pneumonia [10, 11, 23–27].

### Autopsy, microbiological, histological studies, and VAP definitions

Tracheal secretions were collected before autopsy and *P*. *aeruginosa* concentration score was computed as follows: 0: < 3.0 log10 cfu/mL; 1: 3.0–3.9; 2: 4.0–4.9; 3: 5–6; 4: > 6 log cfu/mL. Seventy-six hours after tracheal intubation, the animal was euthanized. We took two samples from the most affected region of each of the five lobes for microbiological assessments. Pulmonary infections were clinically suspected when two of the following clinical signs were present: white blood cell (WBC) ≥ 14,000 per mm^3^, purulent secretion, and body temperature ≥ 38.5 °C. Pulmonary biopsies were evaluated by pathologists and microbiologists blinded to the study treatments, and VAP was confirmed according to a lobar histological injury score ≥ 3 (3 points = pneumonia, 4 points = confluent pneumonia, and 5 points = abscessed pneumonia), associated with a quantitative *P*. *aeruginosa* culture ≥ 3 log cfu/g [19, 28, 29].

### Statistical analysis

A sample size of at least seven animals per group was calculated on the basis of the primary outcome of the original study [18], which was powered to detect a difference in *P*. *aeruginosa* lung tissue concentration between control, EFB + PEEP, and Trendelenburg groups of 3 ± 1.5 log cfu/g, 1 ± 1.5 log cfu/g, and 0 ± 1.5 log cfu/g, respectively, for an assumed effect size of 0.83, a fixed power of 0.85%, and an alpha error probability of 0.05. Restricted maximum likelihood (REML) analysis, based on repeated measures approach, including type of infection, study treatments, and study times, were conducted to evaluate differences in cytokines concentrations. Post-hoc multiple comparisons among groups were computed through Bonferroni adjustment. The area under the receiver operating curves (ROC-AUC) of clinical parameters were computed. Relationship between inflammatory biomarkers and *P*. *aeruginosa* lung tissue concentration was evaluated by linear regression analysis. Statistical analyses were performed using SAS software (version 9.4; SAS Institute, Cary, NC).

## Results

Inflammatory biomarkers of six, eight, and seven pigs—originally randomized in the control, EFB + PEEP, and Trendelenburg groups, respectively—were available for analysis. One pig in the EFB + PEEP group was euthanized earlier for accidental extubation and colonization/histology of the lungs was not examined. Thus, we ultimately analyzed data of six control pigs and seven animals in the EFB + PEEP and Trendelenburg groups. Overall, ten animals developed VAP (four controls, six EFB + PEEP, and zero Trendelenburg). In the control, EFB + PEEP, and Trendelenburg groups, lung *P*. *aeruginosa* burden was 2.4 ± 1.5, 1.9 ± 2.1, and 0.3 ± 0.6 log_10_ cfu/mL, respectively (*p* = 0.020). Whereas, lung bacterial burden was 2.4 ± 1.9 log_10_ cfu/g in animals with VAP, in comparison with 0.6 ± 0.9 in animals without VAP (*p* < 0.001).

### Clinical and microbiology studies

Table 2 reports clinical and microbiology variables among study groups, whereas Table 3 report difference between animals with or without VAP. Among study treatments, the following variables changed significantly: body temperature (37.0 ± 1.6, 38.4 ± 2.1, and 37.1 ± 1.3 °C in the control, EFB + PEEP, and Trendelenburg groups, respectively, *p* < 0.001) and CPIS calculated at 72 h, before autopsy (4.6 ± 0.9, 6.3 ± 0.5, and 4.6 ± 0.9, *p* = 0.035). Whereas, in animals with VAP, *P*. *aeruginosa* concentration in tracheal aspirates was 6.3 ± 0.6 log_10_ cfu/mL, in comparison with 5.4 ± 0.9 in animals without VAP (*p* = 0.024). As for others clinical parameters of organ injury, creatinine (pig reference value 06–1.2 mg/dL) was 1.16 ± 0.38, 1.42 ± 0.45, and 1.40 ± 0.28 mg/dL in the control, EFB + PEEP, and Trendelenburg groups, respectively, (*p* < 0.001); whereas in the animals with VAP was 1.41 ± 0.45 and 1.24 ± 0.31 without VAP was (*p* < 0.001). Finally, alanine aminotransferase (pig reference value 10–40 UI/L) was 41.7 ± 16.4, 34.4 ± 13.9, and 25.8 ± 9.6 U/L in the control, EFB + PEEP, and Trendelenburg groups, respectively, (*p* < 0.001); whereas in the animals with VAP was 31.2 ± 13.8 and 37.3 ± 16.2 U/L without VAP (*p* = 0.914).

**Table 2:** Clinical and microbiology variables among study treatments

Parameter	Time of assessment	Control (6)	EFB + PEEP (7)	Trendelenburg (7)	*p* value
Body temperature (**°**C)	Throughout study time	37.0 ± 1.6	38.4 ± 2.1	37.1 ± 1.3	< 0.001
	72 h	37.5 ± 0.9	39.6 ± 0.8	36.7 ± 1.3	
White blood cells (× 10^9^/L)	Throughout study time	17.2 ± 6.8	13.4 ± 4.3	17.7 ± 5.9	0.133
	72 h	17.3 ± 7.5	12.7 ± 6.1	18.1 ± 2.8	
PaO_2_/FIO_2_ (mmHg)	Throughout study time	424.8 ± 88.9	423.3 ± 76.5	443.6 ± 43.3	0.312
	72 h	378.0 ± 85.0	339.1 ± 26.5	437.0 ± 40.6	
CPIS	72 h	4.6 ± 0.9	6.3 ± 0.5	4.6 ± 0.9	0.035
Tracheal aspirate *P. aeruginosa* quantitative culture (log_10_ cfu/mL)	72 h	5.9 ± 1.1	6.0 ± 0.9	5.6 ± 1.0	0.487

Data are reported as mean ± standard deviation of various assessments throughout the study time or only at 72 h. Per each group, number of studied animals are reported between parenthesis. Of note, we report analyses *p* values of only the values of CPIS and tracheal aspirate *P*. *aeruginosa* quantitative culture at 72 h, whereas for the remaining parameters, we report *p* values of analysis of all assessed parameters throughout the study time (0, 24, 48, and 72 h). CPIS was computed as reported in the Table 1. *PaO*_2_/*FIO*_2_ arterial partial pressure of oxygen/inspiratory fraction of oxygen ratio, *CPIS* clinical pulmonary infection score, *EFB* + *PEEP* expiratory flow bias and positive end expiratory pressure group

**Table 3:** Clinical and microbiology variables between animals with or without VAP

Parameter	Time of assessment	No-VAP (10)	VAP (10)	*p* value
Body temperature (**°**C)	Throughout study time	37.2 ± 1.6	37.9 ± 1.9	0.592
	72 h	37.2 ± 1.6	38.8 ± 1.1	
White blood cells (× 10^9^/L)	Throughout study time	16.8 ± 5.6	15.3 ± 6.4	0.420
	72 h	16.5 ± 4.1	15.2 ± 7.5	
PaO_2_/FIO_2_ (mmHg)	Throughout study time	438.5 ± 58.5	390.5 ± 40.1	0.946
	72 h	430.4 ± 40.5	334.7 ± 47.7	
CPIS	72 h	4.6 ± 0.9	5.8 ± 0.9	0.260
Tracheal aspirate *P. aeruginosa* quantitative culture (log_10_ cfu/mL)	72 h	5.4 ± 1.0	6.3 ± 0.7	0.041

Data are reported as mean ± standard deviation of various assessments throughout the study time or only at 72 h. Per each group, number of studied animals are reported between parenthesis. Of note, we report analyses *p* values of only the values of CPIS and tracheal aspirate *P*. *aeruginosa* quantitative culture at 72 h, whereas for the remaining parameters, we report *p* values of analysis of all assessed parameters throughout the study time (0, 24, 48, and 72 h). CPIS was computed as reported in the Table 1. *VAP* ventilator-associated pneumonia, *PaO*_2_/*FIO*_2_ arterial partial pressure of oxygen/inspiratory fraction of oxygen ratio, *CPIS* clinical pulmonary infection score

### The effects of study treatments on serum inflammatory markers

**Fig. 1:**
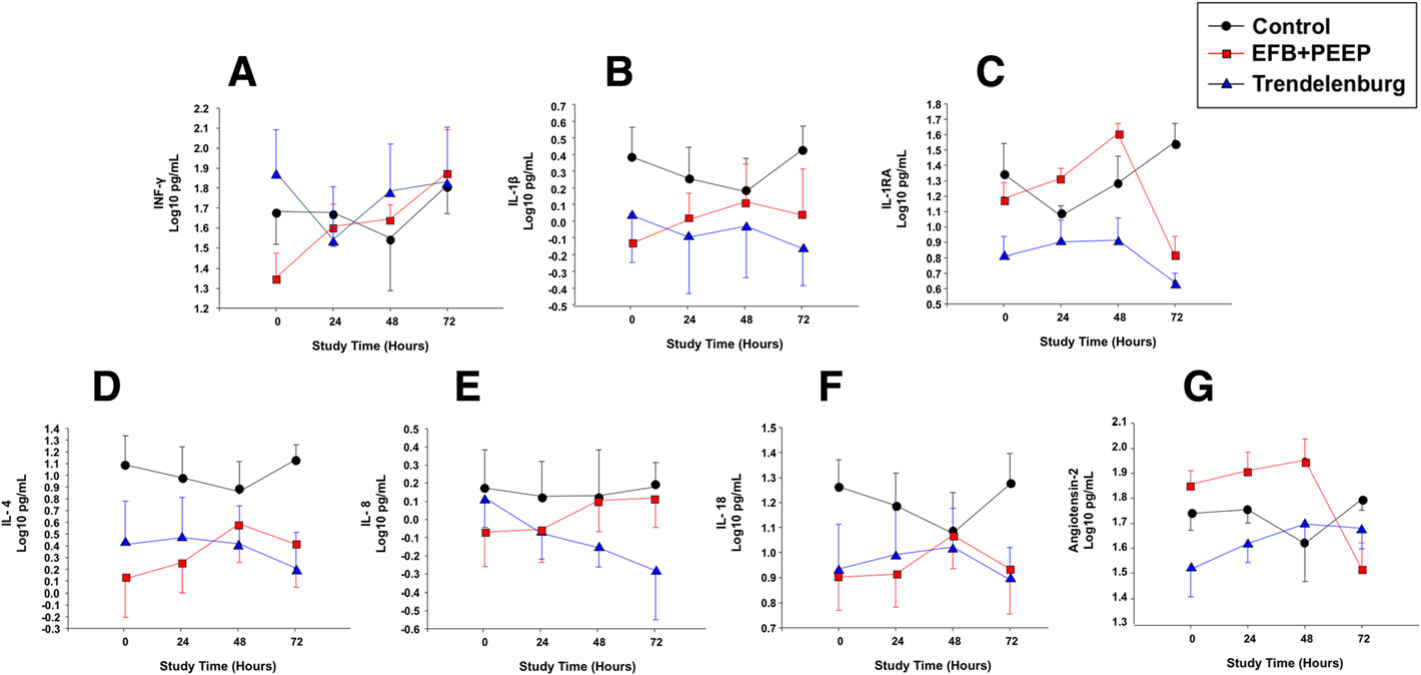
Cytokines that significantly differed among study treatments, per times of assessment. **a** IFN-γ differed among study treatments (*p* = 0.043); no differences were found among times of assessment (*p* = 0.470) and study treatments × times of assessment (*p* = 0.847). **b** IL-1ß differed among study treatments (*p* = 0.038); whereas, we did not find differences among times of assessment (*p* = 0.869) and study treatments × times of assessment (*p* = 0.973). **c** IL-1RA differed among study treatments (*p* < 0.001); whereas, we did not find differences among times of assessment (*p* = 0.151) and study treatments × times of assessment (*p* = 0.618). **d** IL-4 differed among study treatments (*p* = 0.064); no differences among times of assessment (*p* = 0.861) and study treatments × times of assessment (*p* = 0.967) were found. **e** IL-8 differed among study treatments (*p* = 0.066) and no differences among times of assessment (*p* = 0.915) and study treatments × times of assessment (*p* = 0.978) were found. **f** IL-18 differed among study treatments (*p* = 0.005); whereas, among times of assessment (*p* = 0.879), and study treatments × times of assessment (*p* = 0.991) no differences were found. **g** Angiotensin-2 differed among study treatments (*p* = 0.048); whereas, among times of assessment (*p* = 0.552), and study treatments × times of assessment (*p* = 0.949) no differences were found. *IFN* interferon, *IL* interleukin, *EFB* + *PEEP* expiratory flow bias and positive end-expiratory pressure group

As depicted in Table 4, study treatments changed significantly levels of INF-γ (*p* = 0.047), IL-1β (*p* = 0.021), IL-1RA (*p* < 0.001), IL-4 (*p* = 0.005), IL-8 (*p* = 0.008), IL-18 (*p* = 0.050), and angiotensin-2 (*p* = 0.048). In particular, as depicted in Fig. 1, at the end of the study, animals positioned in Trendelenburg presented lower levels of all aforementioned inflammatory markers, but INF-γ and angiotensin-2.

**Table 4:** Inflammatory markers among study groups

Inflammatory marker (log_10_ pg/L)	Control (6)	EFB + PEEP (7)	Trendelenburg (7)	*p* value
INF-ɣ	1.70 ± 0.42	1.44 ± 0.33	1.76 ± 0.59	0.048
IL-1α	− 0.62 ± 0.47	− 0.98 ± 0.54	− 0.80 ± 0.64	0.709
IL-1β	0.35 ± 0.41	− 0.06 ± 0.49	− 0.03 ± 0.72	0.021
IL-1RA	1.33 ± 0.33	1.28 ± 0.31	0.82 ± 0.33	< 0.001
IL-2	− 0.04 ± 0.37	− 0.29 ± 0.51	− 0.25 ± 0.79	0.558
IL-4	1.01 ± 0.52	0.25 ± 0.73	0.38 ± 0.83	0.005
IL-6	− 0.01 ± 0.29	− 0.07 ± 0.42	− 0.15 ± 0.63	0.806
IL-8	0.16 ± 0.45	− 0.03 ± 0.42	− 0.08 ± 0.41	0.008
IL-10	− 0.42 ± 0.45	0.13 ± 0.41	0.21 ± 0.53	0.450
IL-12	0.93 ± 0.13	0.77 ± 0.18	0.74 ± 0.23	0.058
IL-18	1.19 ± 0.31	0.94 ± 0.31	0.96 ± 0.40	0.050
TNF-alpha	− 0.16 ± 0.53	− 0.08 ± 0.48	− 0.31 ± 0.49	0.814
TF	1.95 ± 0.20	1.98 ± 0.16	2.04 ± 0.17	0.232
Angiotensin-2	1.73 ± 0.21	1.87 ± 0.17	1.62 ± 0.23	0.048
ADM	2.79 ± 0.80	2.99 ± 0.24	2.84 ± 0.65	0.515
CRP	4.15 ± 0.39	3.86 ± 0.55	3.55 ± 0.51	0.052

Determinations of interferon-γ (INF-γ), interleukin (IL)-1α, IL-1β, IL-1 receptor antagonist (RA), IL-2, IL-4, IL-6, IL-6, IL-8, IL-10, IL-12, IL-18, tumor necrosis factor (TNF)-α, tissue factor (TF), angiotensin-2, adrenomedullin (ADM), and C-reactive protein (CRP) in serum throughout the study are shown among study groups and in animals with or without VAP. Data are reported as mean ± standard deviation based on log_10_ transformation. *EFB* + *PEEP* expiratory flow bias and positive end expiratory pressure group

### Serum inflammatory markers to diagnose ventilator-associated pneumonia

**Fig. 2:**
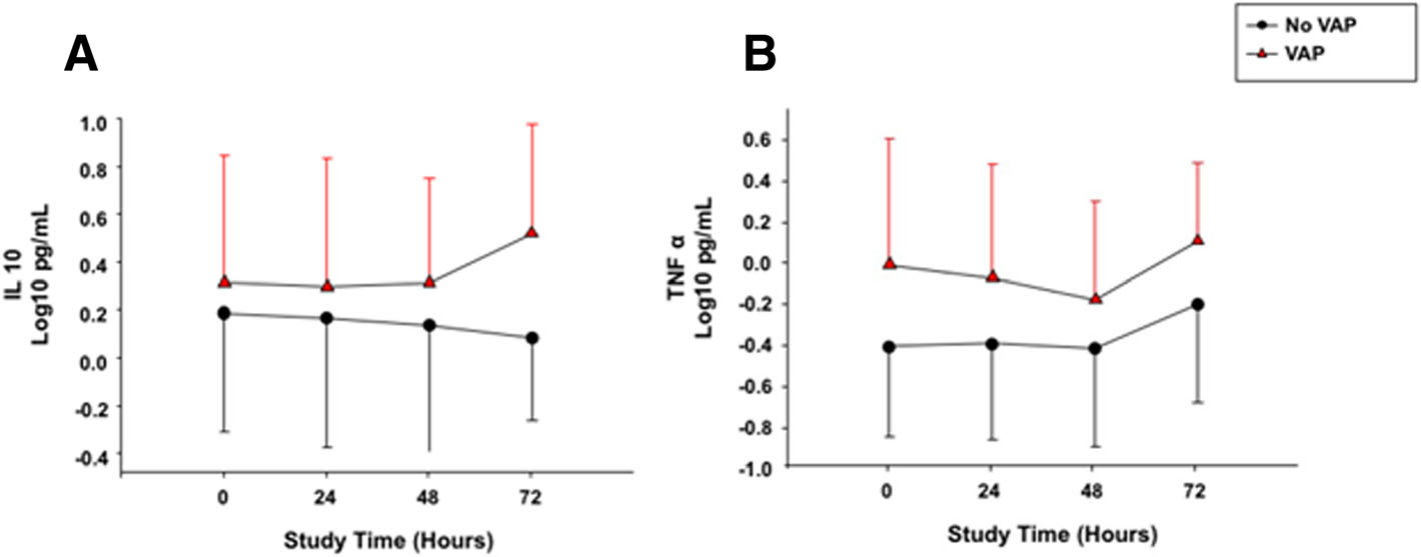
Cytokines that significantly differed between animals with or without VAP, per times of assessment. **a** IL-10 differed among animals with or without VAP (*p* = 0.028) and for occurrence of VAP × study treatments (*p* = 0.029); whereas, among times of assessment (*p* = 0.984) and occurrence of VAP × times of assessment (*p* = 0.999) no differences were found. **b** TNF**-α** differed among types of pulmonary infection (*p* = 0.003) and study treatments (*p* = 0.008); whereas, among types of pulmonary infection × study treatments (*p* = 0.007); times of assessment (*p* = 0.984) and types of pulmonary infection × times of assessment (*p* = 0.995) no differences were found. *IFN*-γ interferon-**γ**, *IL* interleukin

As depicted in Table 5, there were significant differences in the concentrations of IL-10 (*p* = 0.035) and TNF-α (*p* = 0.041) when comparing animals with or without VAP. We report in Fig. 2 dynamics of aforementioned cytokines, among animals with or without VAP.

**Table 5:** Inflammatory markers between animals with or without VAP

Inflammatory marker (log_10_ pg/L)	No-VAP (10)	VAP (10)	*p* value
INF-ɣ	1.66 ± 0.58	1.60 ± 0.35	0.165
IL-1α	− 0.84 ± 0.58	− 0.79 ± 0.57	0.339
IL-1β	0.04 ± 0.67	− 0.12 ± 0.48	0.640
IL-1RA	0.99 ± 0.42	1.28 ± 0.31	0.663
IL-2	− 0.30 ± 0.69	− 0.10 ± 0.47	0.152
IL-4	− 0.58 ± 0.80	− 0.49 ± 0.75	0.130
IL-6	− 0.14 ± 0.53	− 0.01 ± 0.40	0.283
IL-8	0.05 ± 0.51	− 0.01 ± 0.35	0.329
IL-10	0.15 ± 0.47	0.36 ± 0.48	0.035
IL-12	0.77 ± 0.20	0.86 ± 0.19	0.281
IL-18	1.03 ± 0.40	1.03 ± 0.31	0.707
TNF-alpha	− 0.33 ± 0.47	− 0.04 ± 0.50	0.041
TF	2.01 ± 0.15	1.98 ± 0.21	0.517
Angiotensin-2	1.67 ± 0.21	1.81 ± 0.22	0.969
ADM	2.78 ± 0.71	2.99 ± 0.40	0.175
CRP	3.69 ± 0.46	4.04 ± 0.57	0.189

Determinations of interferon-γ (INF-γ), interleukin (IL)-1α, IL-1β, IL-1 receptor antagonist (RA), IL-2, IL-4, IL-6, IL-6, IL-8, IL-10, IL-12, IL-18, tumor necrosis factor (TNF)-α, tissue factor (TF), angiotensin-2, adrenomedullin (ADM), and C-reactive protein (CRP) in serum throughout the study are shown among study groups and in animals with or without VAP. Data are reported as mean ± standard deviation based on log_10_ transformation. *VAP* ventilator-associated pneumonia

**Fig. 3:**
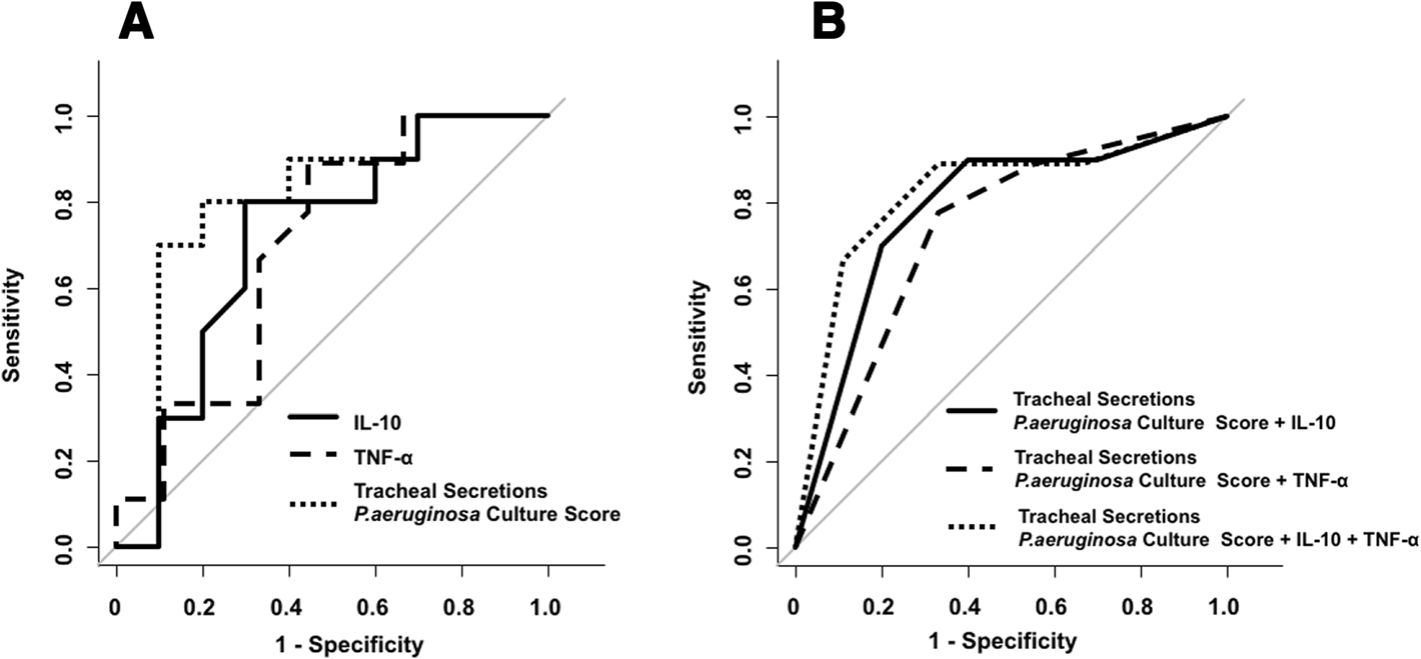
Analysis of the receiver operating characteristics curves. **a** Analysis of the receiver operating characteristic curve for IL-10, TNF-α, and the tracheal secretions *P*. *aeruginosa* concentration score, which was computed as follows: 0 = < 3.0 log_10_ cfu/mL; 1 = 3.0–3.9 log_10_ cfu/mL; 2 = 4.0–4.9 log_10_ cfu/mL; 3 = 5–5.9 log_10_ cfu/mL; 4 = ≥ 6 log10 cfu/mL. The area under the receiver operating characteristics curves of IL-10, TNF-α, and the tracheal secretions *P*. *aeruginosa* concentration score were 0.71, 0.69, and 0.81, respectively. **b** Analysis of the receiver operating characteristic curve for tracheal secretions *P. aeruginosa* concentration score with IL10, TNF-α, or IL10 and TNF-α. The area under the receiver operating characteristics curves of tracheal secretions *P*. *aeruginosa* concentration score with IL10 was 0.78, of tracheal secretions *P*. *aeruginosa* concentration score with TNF-α was 0.73, and of tracheal secretions *P*. *aeruginosa* concentration score with IL-10 and TNF-α was 0.82. We did not find any statistically significant differences among the tested receiver operating characteristics curves

**Fig. 4:**
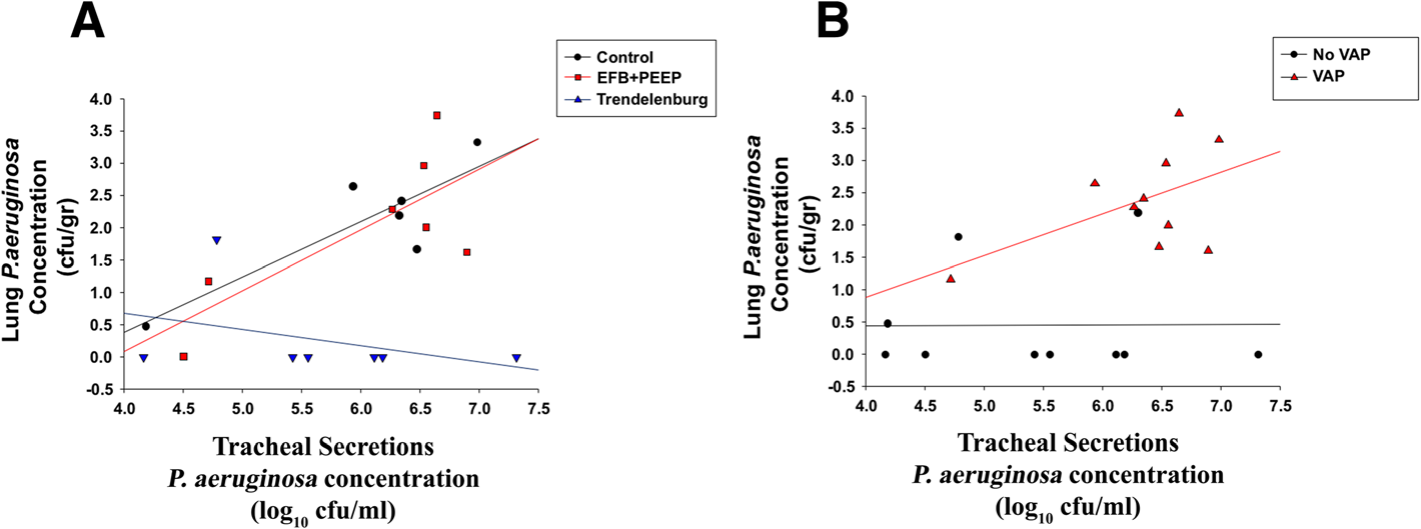
Lung *P*. *aeruginosa* burden as a function of tracheal secretions *P*. *aeruginosa* burden. **a** The linear regression equation was fitted to predict lung *P*. *aeruginosa* burden by tracheal secretions *P*. *aeruginosa* burden (log10 cfu/mL) and clustered by study groups. Regression equation control group: [lung *P*. *aeruginosa* burden (log_10_ cfu/g) = − 3.06 + (0.85 × tracheal secretions *P*. *aeruginosa* burden (log_10_ cfu/mL)]. *N* = 6, *R* = 0.85, *R*^2^ = 0.73, Adjusted *R*^sqr^ = 0.67, *p* value = 0.029. Regression equation EFB + PEEP group: [lung *P*. *aeruginosa* burden (log_10_ cfu/g) = − 3.68 + (0.94 × tracheal secretions *P*. *aeruginosa* burden (log_10_ cfu/mL)]. *N* = 7, *R* = 0.76, *R*^2^ = 0.57, Adjusted *R*^sqr^ = 0.49, *p* value = 0.048. Regression equation Trendelenburg group: [lung *P*. *aeruginosa* burden (log_10_ cfu/g) = − 1.67 + (− 0.25 × tracheal secretions *P*. *aeruginosa* burden (log_10_ cfu/mL)]. *N* = 7, *R* = 0.37, *R*^2^ = 0.14, Adjusted *R*^sqr^ = 0.00, *p* value = 0.411. **b** The linear regression equation was fitted to predict lung *P*. *aeruginosa* burden by tracheal secretions *P*. *aeruginosa* burden (log10 cfu/mL) and clustered by development of ventilator-associated pneumonia (VAP). Regression equation VAP: [lung *P*. *aeruginosa* burden (log_10_ cfu/g) = − 1.69 + (0.64 × tracheal secretions *P*. *aeruginosa* burden (log_10_ cfu/mL)]. *N* = 10, *R* = 0.51, *R*^2^ = 0.26, Adjusted *R*^sqr^ = 0.17, *p* value = 0.130. Regression equation no VAP: [lung *P*. *aeruginosa* burden (log_10_ cfu/g) = 0.41 + (0.007 × tracheal secretions *P*. *aeruginosa* burden (log_10_ cfu/mL)]. *N* = 10, *R* = 0.01, *R*^2^ = 0.00, Adjusted *R*^sqr^ = 0.00, *p* value = 0.980. *EFB* + *PEEP* expiratory flow bias and positive end expiratory pressure group

On the basis of aforementioned findings, the capacity for IL-10 and TNF-α and tracheal secretions *P*. *aeruginosa* burden to diagnose VAP were tested, and ROC curves computed (Table 6 and Fig. 3). We found that the best model, which provided moderate discrimination for the diagnosis of VAP, with a ROC-AUC of 0.82 (95% CI 0.61–1.00) comprised all aforementioned parameters. Linear regression analyses showed that IL-10 (*p* = 0.995), TNF-α (*p* = 0.160), and tracheal secretions *P*. *aeruginosa* burden score (*p* = 0.068) were not significantly associated with lung *P*. *aeruginosa* burden. In Fig. 4, we depicts tracheal secretions *P*. *aeruginosa* burden capability to predict lung burden, clustered by study groups and development of VAP. Of note, in animals that developed VAP, tracheal secretions *P*. *aeruginosa* burden was poorly associated with lung burden (*p* = 0.131).

**Table 6:** Receiver operating curves parameters

	AU-ROC (95% CI)	Best cut-off value*	Sensitivity	Specificity	PPV	NPV
Single VAP diagnostic parameter						
IL-10 (log_10_ pg/L)	0.71 (0.47–0.96)	0.250	80%	70%	73%	78%
TNF-α (log_10_ pg/L)	0.69 (0.44–0.96)	− 0.190	89%	56%	67%	83%
Tracheal secretion *P. aeruginosa* concentration (cfu/mL)	0.80 (0.58–1.00)	6.34	70%	90%	88%	75%
Tracheal secretion *P. aeruginosa* concentration score^a^	0.71 (0.50–0.92)	4	80%	60%	67%	75%
Combined VAP diagnostic parameter^b^						
IL-10 + tracheal secretion *P. aeruginosa* concentration score^a^	0.78 (0.58–0.99)	5	70%	80%	78%	78%
TNF-α + tracheal secretion *P. aeruginosa* concentration score^a^	0.73 (0.51–0.95)	5	78%	67%	70%	75%
IL-10 + TNF-α + tracheal secretion *P. aeruginosa* concentration score^a^	0.82 (0.61–1.00)	6	67%	89%	86%	73%

*Receiver operating curves of ventilator-associated pneumonia diagnostic parameters and their combination. *The optimal cut-off values were computed through the Youden’s index (J), which is the maximal vertical distance between the ROC curve and the first bisector (or chance line)

^a^The tracheal secretion *P*. *aeruginosa* concentration score was computed as follows: 0: < 3.0 log cfu/mL; 1: 3.0–3.9 log cfu/mL; 2: 4.0–5.9 log cfu/mL; 3: 5–6 log cfu/mL; 4: > 6 log cfu/mL. *AU*-*ROC* area under receiver operating curve, *CI* confidence interval, *PPV* positive predictive value, *NPV* negative predictive value, *IL* interleukin, *TNF* tumor necrosis factor

^b^To combine interleukins and tracheal secretion *P*. *aeruginosa* concentration score, we categorized IL-10 and TNF-α as 0–1 values, based on the best cut-off value

## Discussion

In this study, we observed that in pigs, challenged into the oropharynx with *P*. *aeruginosa*, the lateral-Trendelenburg position reduced systemic inflammation through the prevention of VAP. Also, this study demonstrated that in a validated animal model of VAP, serum IL-10 and TNF-α were the only cytokines that varied during VAP development. Yet, culture of tracheal secretions still outperformed all evaluated diagnostic parameters.

### Effects of study interventions on systemic inflammation

We consistently demonstrated in previous studies in sheep [16, 30] and pigs [17, 31] that the Trendelenburg position avoided VAP, but to the best of our knowledge, this is the first comprehensive report regarding its effects on systemic inflammation. Our study adds to previous literature and suggests that during mechanical ventilation, the Trendelenburg position might limit systemic inflammation. In particular, we found that IL-1β, IL-1RA, IL-4, and IL-8 were consistently lower in the Trendelenburg group. In contrast, modifying duty cycle and PEEP did not have any effect on systemic inflammation and, as previously reported [18], on VAP. Importantly, our study primarily focused on cytokines that might variate during the development of VAP, thus it is plausible that the association of aforementioned cytokines with the Trendelenburg position might have been related to the prevention of VAP. Indeed, previous findings confirmed higher levels of IL-1β, specifically in bronchoalveolar lavage fluids [24, 26], of patients with VAP, whereas an association between systemic and pulmonary IL-1RA and VAP [10, 26] has not been established. As for IL-4, this biomarker has not been tested in VAP and preliminary studies have found in IL-4-knockout mice resistance to *P*. *aeruginosa* pulmonary infection and increased TNF-α production [32]. Also in pediatric patients with pneumonia, IL-4 was a reliable marker of severity of the disease [23]. Finally, clinical studies have confirmed a surge in IL-8 with VAP [27].

### VAP diagnosis

Considering that in clinical settings VAP still lacks of a diagnostic gold standard, an additional purpose of our study was assessing accuracy of several diagnostic parameters. In line with previous reports [29], clinical variables, such as body temperature, WBC, and PaO_2_/FIO_2_, were highly unspecific. As for systemic cytokines, previous clinical studies [10, 24, 25, 33–36] have appraised biomarkers in bloodstream, lungs, and pleural space to find the best diagnostic marker. Yet, discriminatory inflammatory markers that could reliably diagnose VAP are difficult to be identified in clinical settings, because at the time of VAP development, ICU patients often present other infections. Furthermore, ICU patients might be in an immune-paralysis state [37–39], which increases the risk of developing VAP [25], while hindering patient’s inflammatory response. Given the abovementioned challenges, the use of a reliable animal model of VAP [28], developed in healthy animals without concomitant illnesses, could facilitate identification of diagnostic markers and redirect on the most promising.

We found that only IL-10 and TNF-α were independently associated with the development of VAP. IL-10 is an anti-inflammatory cytokine that inhibits activation and effector function of T cells, monocytes, and macrophages [40]. Millo and collaborators [26] did not find any variation in plasma and BAL IL-10 in patients who developed VAP. Similarly, Conway Morris et al. confirmed that IL-10 did not have potential value for discriminating VAP from non-infected patients [34]. Whereas, Martin-Loeches and collaborators found significant differences in IL-10 concentration between VAP and no-VAP patients [10]; nevertheless, multivariate analyses failed to corroborate diagnostic value of IL-10. TNF-α is predominately produced by macrophages and exert various effects such as fever, cachexia, and inhibition of tumorigenesis and viral replication. Millo et al. found higher levels of TNF-α in bronchoalveolar lavage fluids of VAP patients [26].

Of note, we found that *P*. *aeruginosa* endotracheal aspirate (ETA) concentration overcame diagnostic accuracy of all cytokines, yielding an AU-ROC higher than 80% (Fig. 3). A clinical trial [41] tested diagnostic value of culture of tracheal secretions vs. bronchoalveolar lavage fluids and it did not find any difference between study groups. Thus, latest European [3] and American [2] guidelines for the management of patients with VAP recommended obtaining samples of respiratory secretions to diagnose VAP. Our findings support this recommendation; yet, it is important to emphasize that tracheal secretions culture requires 1 to 3 days before definitive results, ultimately limiting initial therapeutic options. Also, as reported in Fig. 4, we found a marginal association between *P*. *aeruginosa* ETA concentration and lung colonization. This could be related to the limited number of animals or to specific features of our model; indeed, following oropharyngeal challenge, the animals consistently developed colonization of the proximal airways, irrespective of the subsequent colonization of the lungs and VAP development.

### Clinical implications

Our preliminary findings should be interpreted in light of the potential clinical applications. First, clinical feasibility of inverse-ratio ventilation is challenging, and given the marginal results reported in our latest analysis and previous studies [18], it should not be recommended in clinical settings. Second, in our studies, we failed to find efficacy of PEEP in reducing systemic inflammation or VAP, but these findings are in contrast with a previous clinical study that found lower incidence of VAP in patients ventilated with PEEP of 5 vs. 0 cm H_2_O [42]. This study was discontinued earlier for low recruitment rate, thus future clinical corroborations are essential to further explore the value of PEEP. Third, the recent results of the Gravity-VAP Trial [8] confirmed preventive benefits associated with the lateral-Trendelenburg position, but the study was discontinued earlier, due to a very low incidence of VAP and marginal effects in secondary outcomes. Of note, in the Gravity-VAP trial, the lateral-Trendelenburg position was applied for only 2 days following intubation, and higher safety was reported in patients who did not present pulmonary infiltrates. Considering the positive results from experimental studies [17, 30, 31], but the limitations of the latest randomized trial, risks and benefits of such intervention should be carefully pondered, carefully examining timing and duration of the intervention, which should exclusively be applied to patients who are not intubated for pulmonary causes. Furthermore, pulmonary and systemic inflammation should be monitored. Finally, although our results confirm diagnostic accuracy of ETA, the delay for culture results often causes overtreatment or inappropriate treatment of multi-drug resistant pathogens. Thus, development of novel rapid molecular assays, custom-made for pathogen specific for VAP and for drug resistance genes, are needed. In addition, given the variability in biomarkers concentration among different patient populations and courses of treatment, a comprehensive evaluation of the dynamics of these markers, rather than the absolute cut-off values should be prioritized.

### Study limitations

First, although we conducted a 72-h study, in clinical settings, VAP may develop after several days of MV; therefore, in our model, some pathogenic mechanisms and the inflammatory response could somehow diverge in comparison with the critically ill, ventilated patient. Second, our animals were healthy at the beginning of the study and inflammatory changes were specifically related to the new iatrogenic infection. Nevertheless, in the early phase of the experiment, inflammatory markers could have been affected by the surgical interventions performed during animal preparation. Third, considering the complexity of cytokine signaling pathways in critically ill patients and potential inter-species differences, our results require further validation in humans. Fourth, this was an analysis of animals included in a previous trial [18] and inferences should primary assist for future confirmatory analyses. Fifth, our findings should be discussed critically, because pigs are quadruped and were maintained prone, due to inherent risks of lung dysfunction when maintained in the supine position for prolonged period of times. Patients are normally maintained in the supine semirecumbent position, and the auto-regulation mechanisms in pulmonary and hemodynamic physiology, which may have played a role in our findings, could be different in pigs and humans. Finally, this study did not encompass the entire range of inflammatory biomarkers that could vary during the course of VAP. For instance, due to methodological limitations of the porcine assay, we did not measure procalcitonin, which was evaluated in previous clinical studies [43].

## Conclusions

In conclusion, this experimental study confirms that in an animal model of *P*. *aeruginosa* VAP, the Trendelenburg position hampers systemic inflammation through avoidance of VAP. In addition, in this model, culture of tracheal secretions is a precise method to diagnose VAP, with marginal improvement in diagnostic accuracy when systemic IL-10 and TNF-α are assessed concurrently. Further clinical studies will be necessary to confirm these hypothesis-generating results.

## Abbreviations

BAL: Bronchoalveolar lavage
CFU: Colony-forming unit
ETA: Endotracheal aspirate
ETT: Endotracheal tube
ICU: Intensive care unit
IL: Interleukin
MV: Mechanical ventilation
PEEP: Positive end-expiratory pressure
REML: Restricted maximum likelihood
ROC-AUC: Area under the receiver operating curves
TNF: Tumor necrosis factor
TP: Trendelenburg position
VAP: Ventilator-associated pneumonia
WBC: White blood cells
